# Smart Sensor Based on Multiple Cascaded Michelson Interferometers with a Long-Period Fiber Grating for Recognition of Paracetamol Concentrations in Solutions

**DOI:** 10.3390/s26154689

**Published:** 2026-07-23

**Authors:** Christian Pintor-Tolentino, Juan Castillo-Mixcóatl, Severino Muñoz-Aguirre, Georgina Beltrán-Pérez

**Affiliations:** Facultad de Ciencias Físico Matemáticas, Benemérita Universidad Autónoma de Puebla, Avenida San Claudio y 18 sur, Col. San Manuel CU, Puebla 72570, Mexico; pt202141695@egresados.buap.mx (C.P.-T.); juan.castillo@correo.buap.mx (J.C.-M.); smunoz@fcfm.buap.mx (S.M.-A.)

**Keywords:** refractometric sensor, cascaded Michelson interferometers, optical fiber, paracetamol, projection to latent structures, support vector machine, projection to latent structures regression

## Abstract

This work introduces a refractometric sensor based on multiple cascaded Michelson interferometers fabricated with long-period fiber gratings to classify different commercial brands of paracetamol as well as to measure solutions with different paracetamol concentrations. The operating principle is based on the interaction of light with the fiber–medium interface, such that variations in the refractive index provoke changes in the spectrum profile that can be measured at the output of the device. The brands used were Medley, Mejoral 500, Tempra, Tylenol, and excipient-free paracetamol (EFP). A support vector machine (SVM) classifier was used to recognize each brand with an F1-score of 92%. Also, using a projection to latent structures regression (PLSR), it was possible to quantify the paracetamol concentration with the best limit of detection (LOD) of 1.58 g/kg for the Mejoral brand.

## 1. Introduction

Nowadays, it is important to investigate the quality of widely used medicines, as well as those that, due to their specific characteristics, may have adverse effects on human health. Several studies have been conducted on paracetamol tablets due to their easy access and increased use, for example, during the COVID-19 pandemic. There are different studies such as the uniformity of unit doses by weight variation, in which the variability of weight per tablet was measured, studies of the solubility of different paracetamol tablets using standard methods in solvents such as distilled water, ethanol, propylene glycol, and glycerin, as well as degradation kinetics using high-performance liquid chromatography, where notable variations were observed as a result of different excipient compositions in the formulas and pharmaceutical technology [1,2,3,4,5,6,7]. In addition, methods have been developed to crystallize the active pharmaceutical ingredients (APIs) of paracetamol for isolation and purification [8,9,10]. Many of these studies have been performed using optical fiber sensors [11,12,13].

On the other hand, refractometric sensors based on long-period fiber gratings (LPFGs) represent a promising technology for detection in various applications, as they offer advantages in terms of sensitivity, size, flexibility, and durability. These types of sensors have been used to detect the salinity level of water, urea, pH, glucose, ammonia, *E. coli*, the crystallization process of paracetamol, among others [14,15,16,17,18]. LPFGs are intrinsically sensitive to changes in the surrounding refractive index, which has enabled their use in the development of sensors based on Michelson and Mach–Zehnder interferometers, widely recognized for their high precision and versatility in multiple applications. In the literature, some works have been reported that employ LPFG-based interferometric sensors for sensing changes in refractive index and detecting chemical compounds in liquid solutions, demonstrating their high sensitivity and stability. For instance, Swart et al. demonstrated the feasibility of using the LPFG-based Michelson interferometer to measure the refractive index and concentration of glycerin mixtures, as well as the immersion depth of the sensor in a liquid and its temperature sensitivity, while Zhuo et al. and Kim et al. used a similar configuration to measure different concentrations in glycerin and glucose solutions, respectively, achieving high system resolution [19,20,21,22]. Moreover, Murphy et al. [23] demonstrated that it is possible to multiplex independent LPFG-based Mach–Zehnder interferometers by observing the phase changes in the fringe patterns due to changes in the refractive index and temperature of Cargille oil. In a previous work [24], we showed the effectiveness of this type of sensor for detecting acetone, which is a biomarker of diabetes mellitus. However, to the best of our current knowledge, the application of LPFG-based sensors for measuring concentration in paracetamol solutions has not been previously reported. Furthermore, machine learning (ML) techniques have not been explored enough for processing the data obtained with LPFG-based sensors. The tools provided by ML for analyzing information are mathematical models that transform complex information into easy-to-interpret data, finding hidden relationships in the original information and discriminating between relevant elements within the data [25,26]. In particular, different ML approaches have been applied to spectral data processing, including methods such as projection to latent structures (PLS), PLS regression (PLSR), support vector machines (SVM) and artificial neural networks (ANN), which have shown usefulness in tasks of classification, prediction and quantitative analysis of such spectral data [27,28,29,30,31,32]. In previous works, we have also shown the effectiveness of SVM applied to this kind of sensor [33,34].

In this study, a smart refractometric LPFG sensor was developed to identify four commercial pharmaceutical brands and to determine (by regression) the API and/or excipient concentration in a solution created using 500 mg tablets for each pharmaceutical brand. The commercial pharmaceutical brands analyzed were Medley, Mejoral 500, Tempra, and Tylenol, as well as excipient-free paracetamol (EFP). This refractometric sensor consisted of four Michelson interferometers in series with different arm lengths and based on four different LPFGs as splitter devices. The interferometric output signal allowed us to observe notable variations in the measured spectral profiles due to the different concentrations and compositions of paracetamol and excipients in the brands studied, respectively. The data were analyzed using PLS as a technique for dimensionality reduction and feature extraction. The use of this technique was necessary to perform the recognition of the different brands, taking advantage of the information contained in the complete spectrum, a task that is not possible to achieve with the traditional demodulation technique. The excipient and paracetamol concentrations were quantified using regressions (PLSR) with the selected latent structures (LSs). The SVM algorithm was used for the recognition task. The results from this sensor showed that it was possible to recognize each brand of paracetamol with an F1-score of 92%, while the best quantification of paracetamol concentration was achieved with an LOD of 1.58 g/kg for the Mejoral brand and an LOD of 1.93 mg for the quantification of the excipient in the Tempra brand.

## 2. Theory

### 2.1. Michelson Interferometer

**Figure 1:**
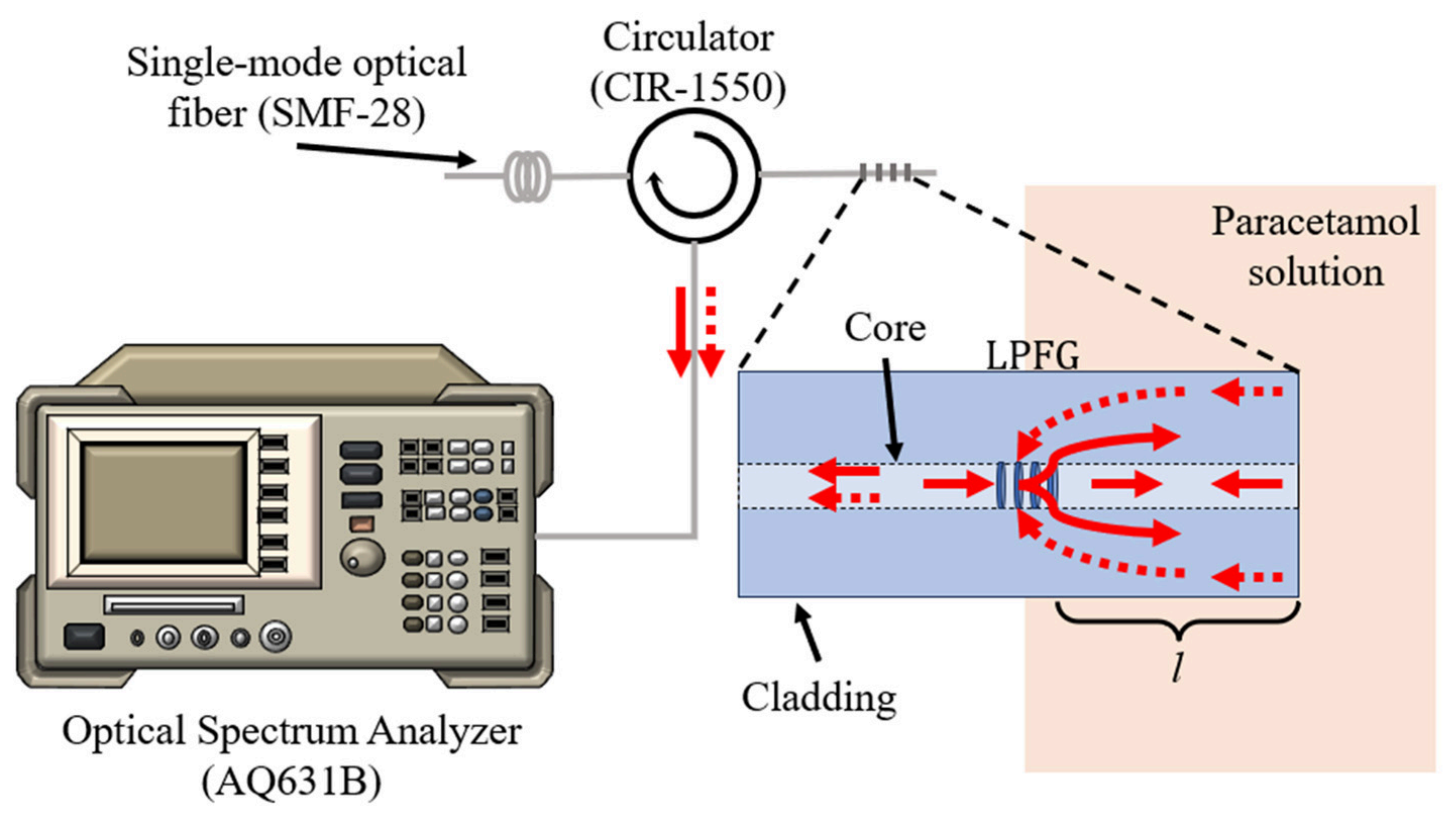
Michelson interferometer based on LPFG.

An optical fiber refractometer sensor is an optical device designed to measure changes in the refractive index of a medium [35,36]. Its operating principle is based on the interaction of light with the fiber-medium interface, such that variations in the refractive index provoke changes in the intensity, phase, or wavelength of the detected optical signal [35], which means a change in the spectrum profile that can be measured at the output of the device. These kinds of sensors are widely used in chemical, biomedical, and environmental applications due to their high sensitivity, immunity to electromagnetic interference, and potential for integration into optical fiber systems [37,38]. In this work, we propose the use of a Michelson interferometer in an optical fiber by using an LPFG as a beam splitter [21,39] as shown in Figure 1. The system works as follows: the fundamental mode of the core travels through the LPFG, which couples part of the energy to a cladding mode, resulting in two beams, one traveling essentially in the core and the other one in the cladding [40]. Both beams are reflected at the fiber tip (boundary with the external medium) due to the difference in refractive indices [21]. These beams travel again through the LPFG in backward propagation in the core (solid and dotted lines), generating interference [39]. The circulator was used to direct these beams toward the optical spectrum analyzer (OSA).

A simple estimation of the interference spectrum can be given as follows: the amplitude of the reflected waves depends on Fresnel reflection (Equation (1)) at the monitored fiber–solution interface [13].

(1) $$R_{c,cl}={\left(\frac{n_{t}(c_{p,e})-n_{c,cl}^{eff}}{n_{t}(c_{p,e})+n_{c,cl}^{eff}}\right)}^{2},$$

where $n_{c,cl}^{eff}$ corresponds to the effective refractive index of the core and cladding of the optical fiber, respectively, and $n_{t}(c_{p,e})$ corresponds to the refractive index of liquid solutions with different concentrations of paracetamol and/or excipient content.

**Figure 2:**
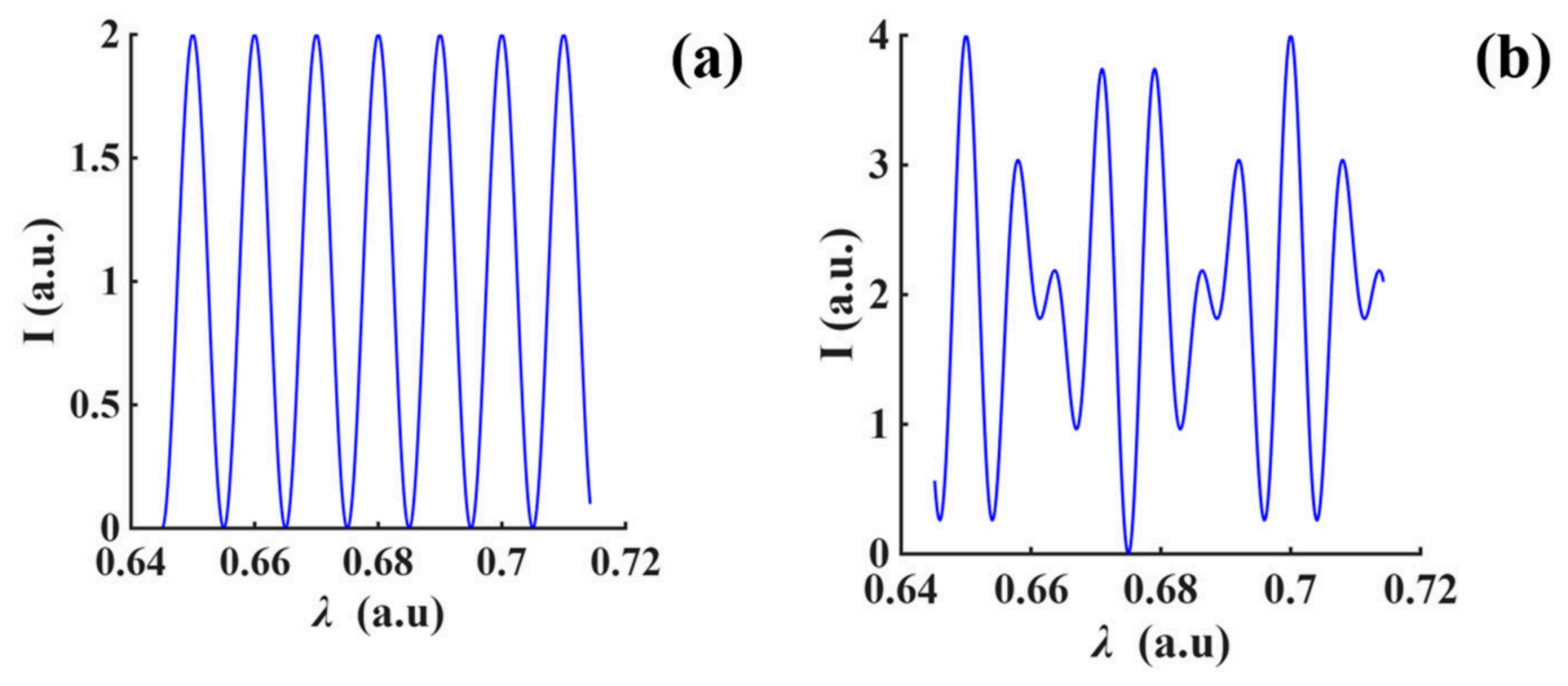
Interferometric intensity as a function of wavelength λ. (**a**) Ideal intensity pattern for a single interferometer. (**b**) Ideal intensity pattern for two different interferometers.

On the other hand, an LPFG is a wavelength-selective filter whose transmission spectra exhibit several resonance peaks resulting from the coupling between the core mode and the different co-propagating cladding modes at wavelengths that obey the resonance condition of Equation (2) [35].

(2) $$\lambda_{res}=\left(n_{c}^{eff}-n_{cl,m}^{eff}\right)\Lambda ,$$

where $\lambda_{res}$ represents the resonance wavelengths, Λ the grating period, and $n_{c}^{eff}$ and $n_{cl,m}^{eff}$ are the effective refractive indices of the core and m-th cladding modes, respectively. The electric field intensity at the circulator output can be evaluated as the superposition of two waves whose intensity can be evaluated using Equation (3) [39].

(3) $$I=E_{1}^{2}+E_{2}^{2}+2E_{1}E_{2}cos\left(\phi_{1}-\phi_{2}\right),$$

where $\phi_{1,2}$ represents the phases of each beam. The phase difference can be evaluated as in Equation (4) [39].

(4) $$\phi_{1}-\phi_{2}=(2\pi /\lambda )\left(n_{c}^{eff}-n_{cl,m}^{eff}\left(c_{p,e}\right)\right)l,$$

where λ is the wavelength, l is the length of the interferometer arms, and $n_{cl,m}^{eff}\left(c_{p,e}\right)$ is the effective index of the m-th cladding mode, which must also be affected by variations in the refractive index of the external medium. In this way, the interference pattern can be affected by amplitude (Equation (1)) and phase variations (Equation (4)). The term l/λ increases the sensitivity of the sensor based on this interferometer. Furthermore, it is possible to place another interferometer of this type by placing another LPFG, with a different period, in cascade with the first one and at a different distance l. If we add more interferometers, we should consider the interference of 2N beams [39] where N is the number of interferometers. However, since in our case the LPFGs are different, for simplicity we considered that the total intensity was the simple superposition of patterns of the type in Equation (3), and we would expect a new pattern with a more complex profile. Figure 2a shows the ideal interference pattern for a single interferometer, while Figure 2b shows the intensity pattern of the superposition of two patterns with different l values. The profile of the latter must change in a more complex way than the former in function of changes in the refractive index of the external medium.

In general, we can expect an interference pattern with an intensity described by Equation (5).

(5) $$I_{T}=\Sigma_{i=1}^{N}I_{i}.$$

**Figure 3:**
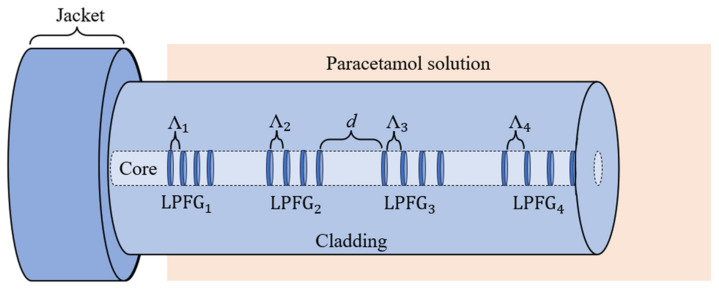
General diagram of the refractometer sensor head with four different LPFGs.

In this work, we propose a sensor consisting of four Michelson interferometers (N=4) formed by different LPFGs in cascade with different lengths l (see Figure 3), to generate a complex interference pattern, which would be a particular fingerprint for each commercial pharmaceutical brand that allows the recognition and quantification of the paracetamol brands.

### 2.2. Machine Learning Techniques

To analyze the interferometric patterns, PLS was used, which is a supervised multivariate analysis method used to model the relationship between a set of predictor variables X and one (or several) sets of response variables Y. PLS reduces the dimensionality of the data using latent variables, constructed in such a way as to maximize the covariance between X and Y, which makes it particularly useful when there are many variables with high collinearity and a limited number of samples [41,42]. Particularly, in the present work, the PLS-NIPALS algorithm was used, whose steps are shown below [41]:

**a**. We defined a starting vector u, that in our case was a vector y with the API (or excipient) concentrations measured related to the respective spectrum, which was also equal to Y, as shown in Equation (6).

(6) u=y.

**b**. Then, the weights w of the matrix X were calculated using Equation (7).

(7) $$w=\frac{X^{\prime }u}{u^{\prime }u}\;,$$

These vectors are intended to help to find the LSs that are most correlated with the values of Y.

**c**. The next step was to project the matrix X onto the vector w to generate the first LS, t of X, as shown in Equation (8).

(8) t=Xw.

**d**. From the vectors t, the weights c of the matrix Y were calculated from Equation (9).

(9) $$c=\frac{Y^{\prime }t}{t^{\prime }t}\;.$$

**e**. The vector u was updated as shown in Equation (10).

(10) $$u=\frac{Yc}{c^{\prime }c}\;.$$

**f**. Then the convergence verification was performed, i.e., the current LS was compared with the LS calculated in the last iteration.

(11) $$\frac{\left\|t_{old}-t_{new}\right\|}{\left\|t_{new}\right\|}<\epsilon ,$$

where ϵ commonly has a value between $10^{-6}$ and $10^{-8}$. If this relationship is not fulfilled, it is necessary to go back to step b until it is satisfied.

**g**. The X and Y matrices were deflated, and they were used as X and Y to calculate the following structure.

(12) $$X_{new}=X{-t}_{new}p^{\prime }$$

(13) $$Y_{new}=Y{-t}_{new}c^{\prime }$$

where p is the X-loading vector, defined in Equation (14).

(14) $$p=\frac{X^{\prime }t}{t^{\prime }t}\;.\;$$

**h**. Each time a useful vector t was found, it was defined as the new LS and stored in a matrix T. Similarly, the weight vector c, and the load vector p, were stored in matrices C and P respectively. This process was repeated until the desired number of LSs was reached.

Once the necessary LS has been chosen, a regression can be performed with this LS (PLSR) to quantify the independent variable (Y). On the other hand, with the same LSs, a classification algorithm can be used to recognize the analyzed brands.

**Figure 4:**
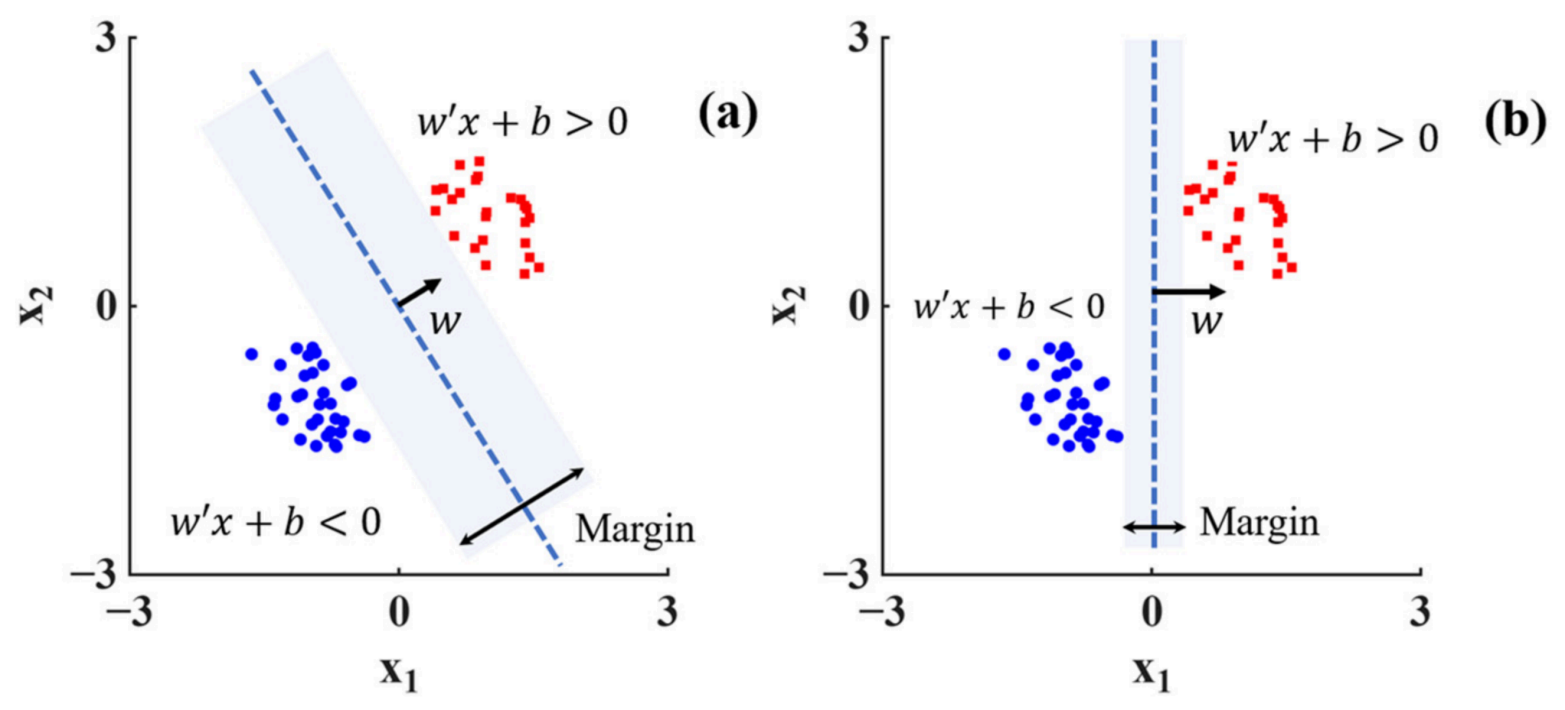
Graphical representation of the SVM showing the separation of two different data classes by a hyperplane (n=2). (**a**) Optimum w value, (**b**) non-optimum w value.

SVM is a supervised learning method widely used in classification problems. The general idea behind SVM is to construct a hyperplane to split a set of data [43,44]. The hyperplane is written as Equation (15).

(15) $$w^{\prime }x+b=0,$$

where w (a vector with n×1 elements) and constant b are the hyperplane parameters and x is a vector with n features values (n×1). For simplicity, Figure 4 shows a graphical representation of the SVM with two different cases of hyperplanes that separate two different kinds of data sets (n=2). As can be seen, the blue margin width determines the SVM performance to correctly classify each class. The main target of SVM is to optimize w (a vector normal to the hyperplane) and b values to maximize the margin width; it can be shown that this optimization can be reached when ${\left\|w\right\|}^{2}/2$ is minimum and subject to $t\left(w^{\prime }x+b\right)\geq 1$, where t is the class label (−1 or 1), for each class respectively [45].

Classification could be done by just evaluating the sign of the hyperplane:

(16) $$w^{\prime }x+b>0,class:+1\;(\mathrm{squares})\;w^{\prime }x+b<0,class:-1\;(\mathrm{balls})$$

**Figure 5:**
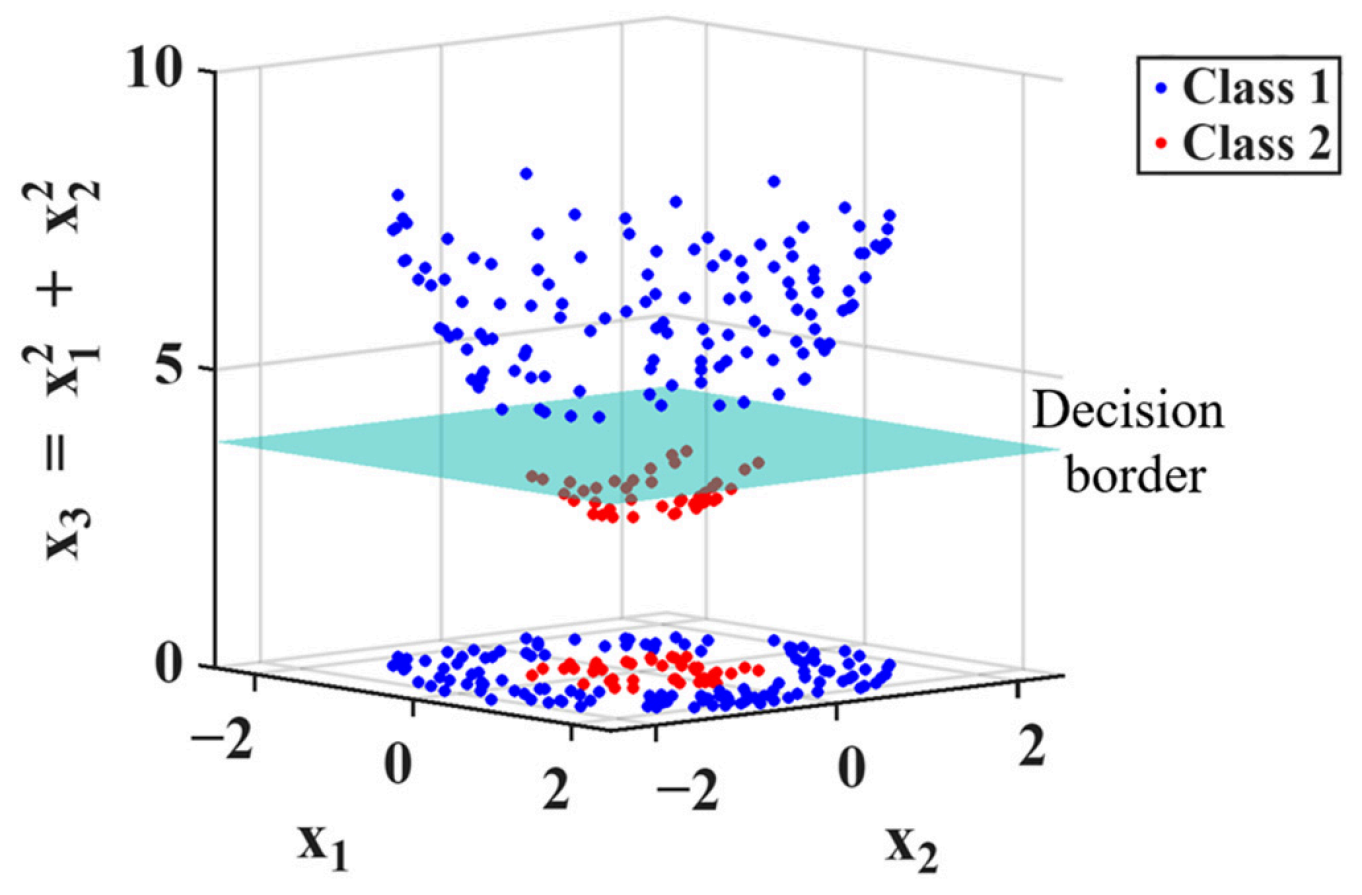
Non-linearly separable data in 2D $\left(x_{1},x_{2}\right)$. The kernel trick allows classification by including new dimensions $\left(z_{1},z_{2},z_{3}\right)=\left(x_{1},x_{2},x_{1}^{2}+x_{2}^{2}\right)$.

The vectors that determine the boundaries of the margin are called the support vectors. Another interesting characteristic of SVM classifiers is the possibility to use the so-called kernel trick. When data are not linearly separable, it is still possible to perform classification if some high-dimensional information is added to the original data. Figure 5 shows a graphical representation of the separation of data using the kernel trick for the case when n=2. Data in the original space ${(x}_{1},x_{2})$ are not linearly separable, however, in the space ${(z}_{1},z_{2},z_{3})=\left(x_{1},x_{2},x_{1}^{2}+x_{2}^{2}\right)$ this separation is possible by the hyperplane shown on the figure. All this can be done without changing the original optimization strategy and this is one of the reasons why SVM is a successful machine learning algorithm.

To quantify the performance of the SVM model, a confusion matrix was used, in which true positives/negatives (TP/TN) and false positives/negatives (FP/FN) were recorded graphically. Based on these values, it is possible to calculate metrics such as accuracy, precision, recall, specificity and F1-score, which allow an objective quantification of the SVM model’s ability to correctly classify data.

**Figure 6:**
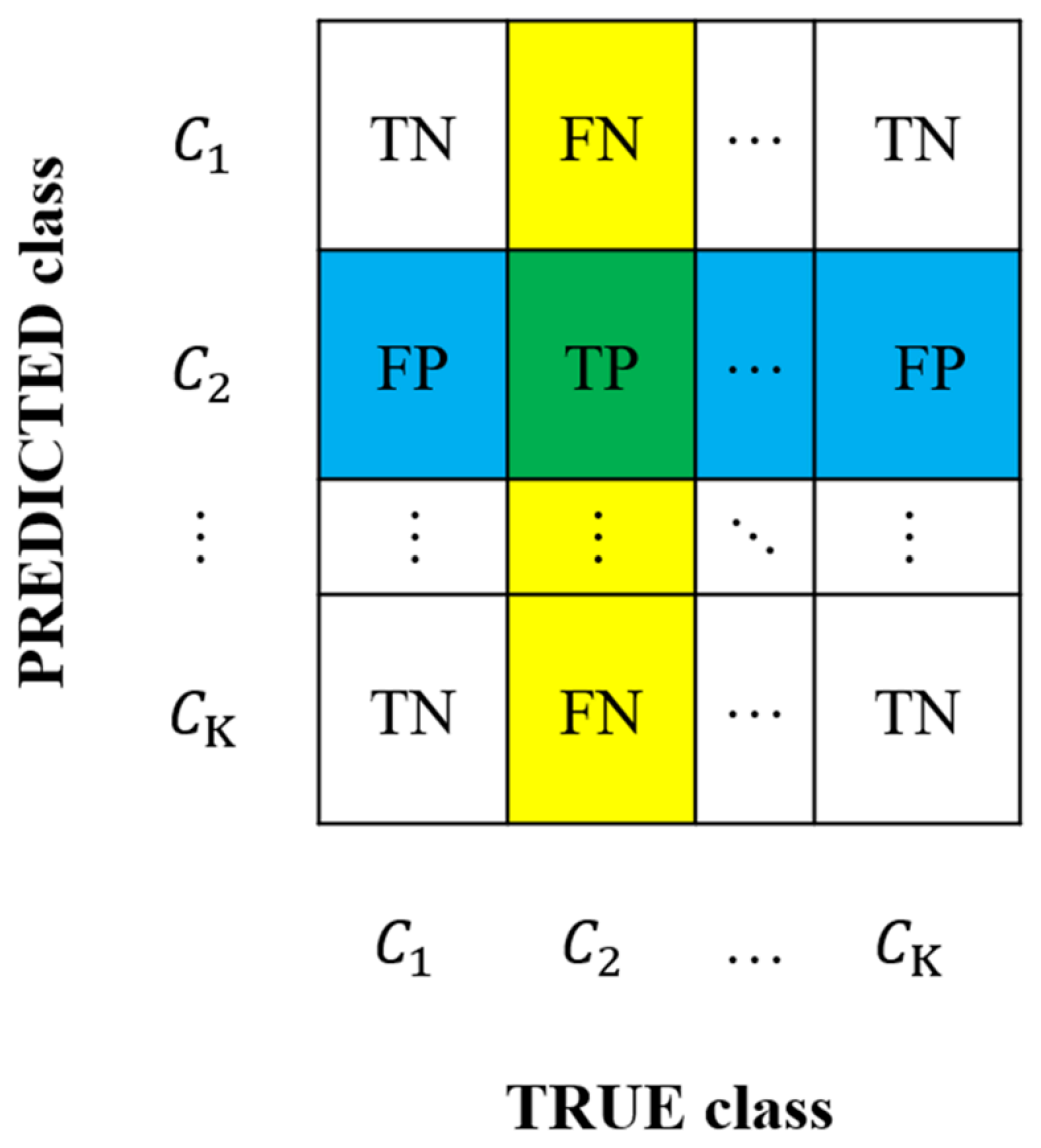
Multiclass confusion matrix.

For multi-class classification, the metrics defined for binary problems cannot be applied directly. The confusion matrix for multiple classes has a K×K dimension, where K corresponds to the number of classes $\left(C_{1},C_{2},\ldots ,C_{K}\right)$. In this context, the traditional definition of TP, TN, FP, FN loses its overall meaning, so the analysis must focus on each class individually, following the characterization shown in Figure 6. Using this approach, it is possible to obtain metrics per class and then combine them appropriately to obtain overall indicators of model performance [46].

Here, the rows correspond to the predicted class, and the columns correspond to the true class. The cells on the diagonal correspond to measurements that have been classified correctly (TP, green square). The cells outside the diagonal correspond to incorrectly classified measurements. In particular, the row of one of the classes (except for the cell on the diagonal) represents the FP (blue squares) of the classifier, as it indicates the measurements that were predicted to belong to that class, but whose true class is different. The column represents FN (yellow squares), i.e., observations whose true class is that class but were classified as another. TN (white squares) are obtained by considering measurements that do not belong to that class and were not classified as such. Taking these quantities into account, it is possible to calculate the model performance metrics as shown in Table 1.

To quantify concentration levels, PLSR was used, which is a specific application of PLS focused on regression problems. In PLSR, the latent variables obtained through PLS are used directly to make a regression model capable of predicting one or more response variables. In this way, PLSR combines dimensionality reduction and regression model estimation in a single procedure, maintaining its robustness against collinearity and noise in the data [41].

**Figure 7:**
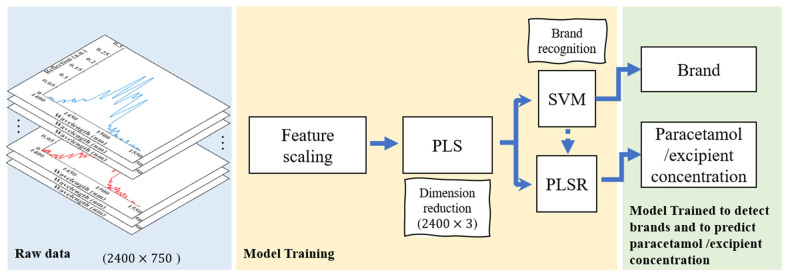
Diagram of the system for recognizing the brand and quantifying the concentration of paracetamol (or excipient) in a solution.

Figure 7 shows the general scheme for brand recognition and quantification of paracetamol and/or excipient present in a solution, which is divided into three stages: (1) The use of PLS for dimensionality reduction and extraction of relevant features from the spectra; an appropriate number of LSs was chosen. (2) Recognition of the drug brand: with the selected LSs, an SVM algorithm was trained to recognize the drug brand under analysis. Finally, (3) the use of PLSR for the quantification of the concentration of paracetamol. Once the drug brand has been determined, the corresponding PLSR model was used to quantify the concentration of paracetamol and/or excipient.

### 2.3. Experimental Setup to Record LPFGs

**Figure 8:**
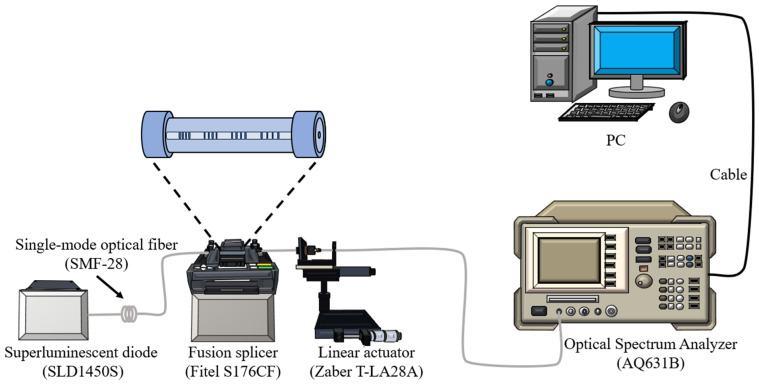
Experimental setup for the LPFGs recording.

Figure 8 shows the experimental setup to record LPFGs. A single-mode optical fiber (SMF-28) with a total length of 80 cm was used. The central section of 10 cm of fiber was stripped and cleaned with ethanol to remove any residue or contaminants. One end of the fiber was then spliced to a superluminescent diode (SLD1450S, Thorlabs, Newton, NJ, USA), which operates in the spectral range from 1430 nm to 1530 nm. The other end of the fiber was spliced to an optical spectrum analyzer (OSA, Ando AQ6315A model 9057 F/8, Tokyo, Japan) to measure the transmission spectrum. A linear actuator (TLA28A, Zaber, Newton, NJ, USA) with a resolution of 1 µm was used for the LPFG recording process. The arc of a splicer was used to generate variations in the refractive index of the fiber, which we call a point. Four LPFGs of 15 points each were recorded with different periods (separation between points). The LPFGs were recorded with a separation of 2 cm and modulation periods of 450 µm, 475 µm, 500 µm, and 525 µm, respectively. The tip of the fiber was cleaved using an optical fiber cutter to ensure reflection.

**Figure 9:**
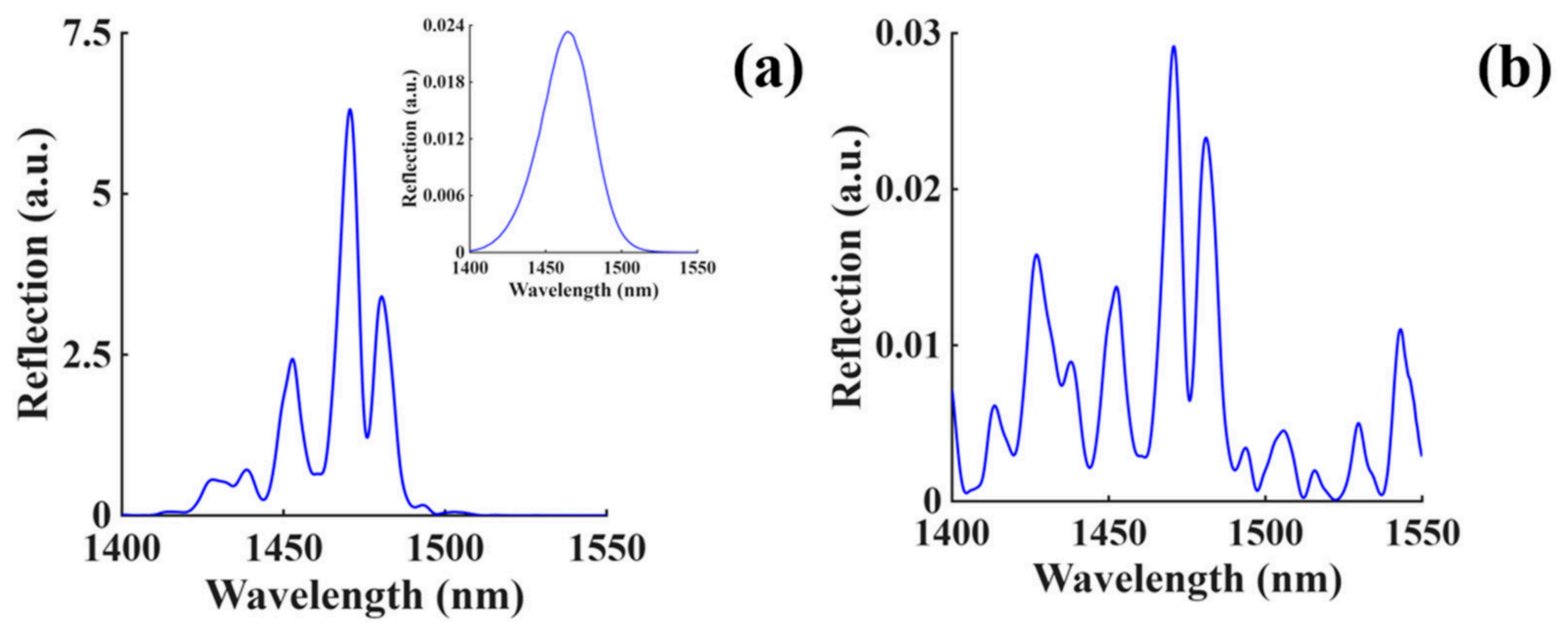
Experimental reflection spectrum of the sensor: (**a**) raw data (the source spectrum is shown in the inset), (**b**) without the light source contribution.

In Figure 9a, we show the raw data of the experimental reflection spectrum of the constructed sensor, and in the inset we show the spectrum of the superluminescent diode as the light source. The normalized reflection spectrum without the source spectrum contribution is shown in Figure 9b. From this figure, we can note that the spectrum resembles the one shown in Figure 2b with a complex profile, which, as was mentioned before, can be used to identify the fingerprints for each commercial pharmaceutical brand analyzed in this work.

### 2.4. Paracetamol Solutions Preparation

The procedure for preparing the solutions was carried out to obtain concentrations of 150, 200, 250, and 300 g/kg for the commercial brands Medley, Mejoral, Tempra, Tylenol, and EFP. According to the information provided by the manufacturers, each tablet of these brands contains 500 mg of API, with an undetermined amount of excipient.

First, an analytical balance was used to determine the individual mass of each tablet and, subsequently, the total mass of the groups corresponding to each concentration. Assuming a content of 500 mg of API per tablet, it was possible to estimate the amount of excipient present, denoted as $m_{i}=\;API+Excipient$.

Next, each group of tablets was pulverized in a ceramic mortar, and the resulting powder was transferred to the container used to prepare the solution. The results obtained are summarized in Table 2.

The solutions with and without paracetamol were prepared at room temperature. A mixture of distilled water and ethanol in a ratio of 60%/40% (m/m) was added to the solute. Considering a total solvent mass of 10 g, this is equivalent to 6 g of water and 4 g of ethanol. The resulting mixture was stirred for 10 min to promote homogenization, obtaining, for example, a solution with a concentration of 1500 mg/10 g (solute/solvent), equivalent to 150 g/kg.

For control solutions, i.e., those prepared without paracetamol, the same proportions of distilled water/ethanol and the same stirring time were used. The purpose was to verify the sensor’s ability to distinguish between samples with and without paracetamol and, additionally, to facilitate the removal of any residue accumulated on the sensor after each measurement.

In the case of EFP (purchased from Sigma-Aldrich (St. Louis, MO, USA), CAS: 103-90-2, with a purity level of 99%), the required amount of API was measured directly using the analytical balance. The solutions with and without paracetamol were prepared using the same proportions of distilled water/ethanol and under the same experimental conditions applied to the commercial brands, following the process described above.

### 2.5. Solutions Measurement

**Figure 10:**
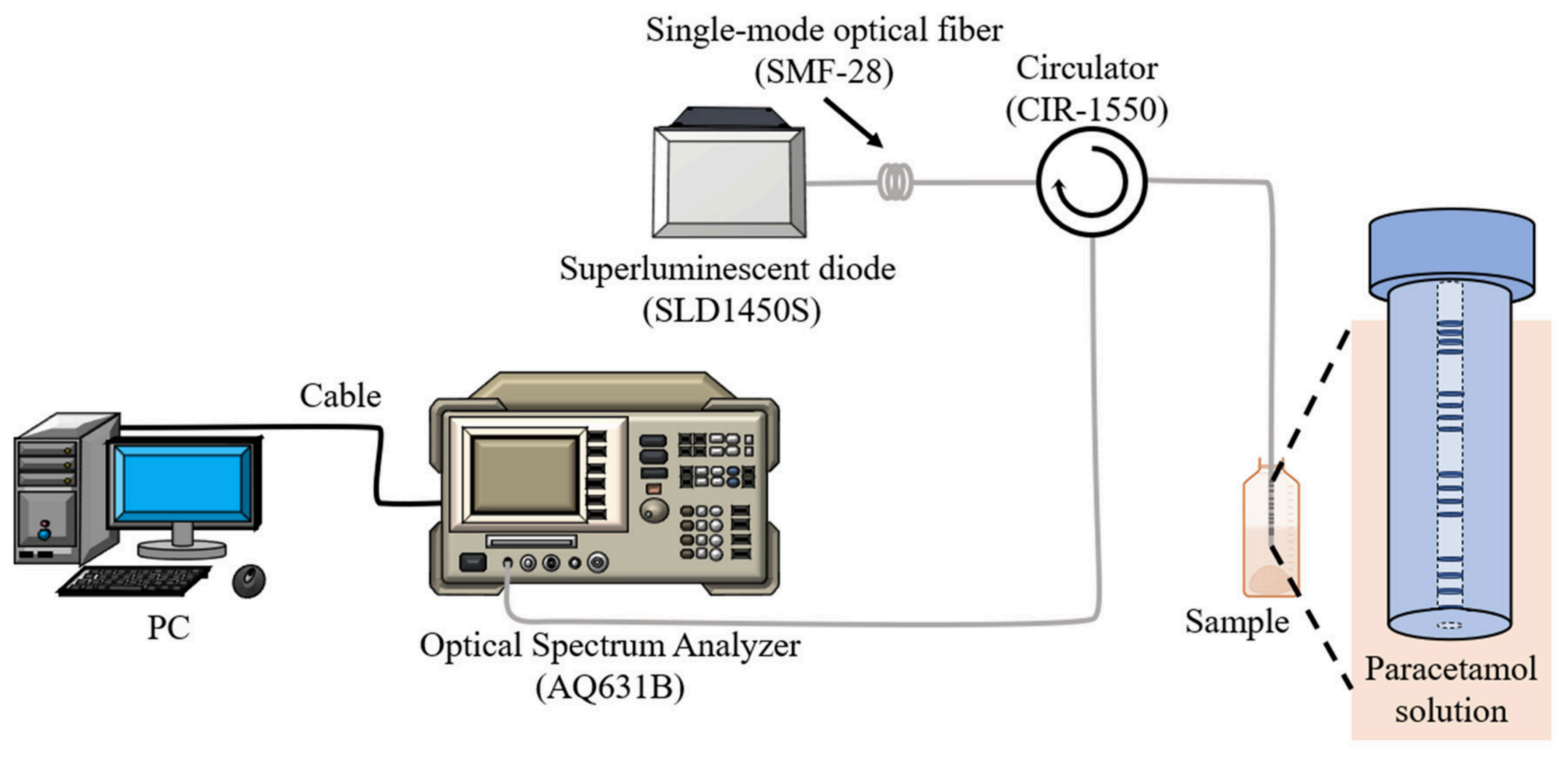
Experimental setup to measure the reflection spectrum of the sensor head for paracetamol/control solution.

Once the sensor was fabricated, it was integrated into an experimental setup for paracetamol/control solution measurement. The experimental setup consisted of an SLD connected to the first port of an optical circulator; the second port was connected to the sensor head, and the third port was connected to an OSA, for measuring the reflectance spectra of each solution prepared for all brands studied in this work (see Figure 10).

Once the experimental setup was ready and the solutions were prepared, the sensor head was placed inside a solution with a concentration of 150 g/kg for a period of 20 min. During this interval, the OSA acquired one reflection spectrum per minute. At the end of the measurement, the sensor was immersed in a control solution under the same experimental conditions, again obtaining one spectrum per minute for 20 min. This allowed the sensor response to be verified in the absence of paracetamol and, at the same time, facilitated the removal of any residues that may have accumulated on the sensor head during the previous measurement. This procedure was repeated for each of the concentrations evaluated (200 g/kg, 250 g/kg, and 300 g/kg), reusing the sensor in each of the measurements, obtaining a set of three measurements per concentration (240 spectra for each brand) and other set of three measurements corresponding to their respective controls (1200 spectra), giving a total of 2400 spectra.

## 3. Results and Discussion

**Figure 11:**
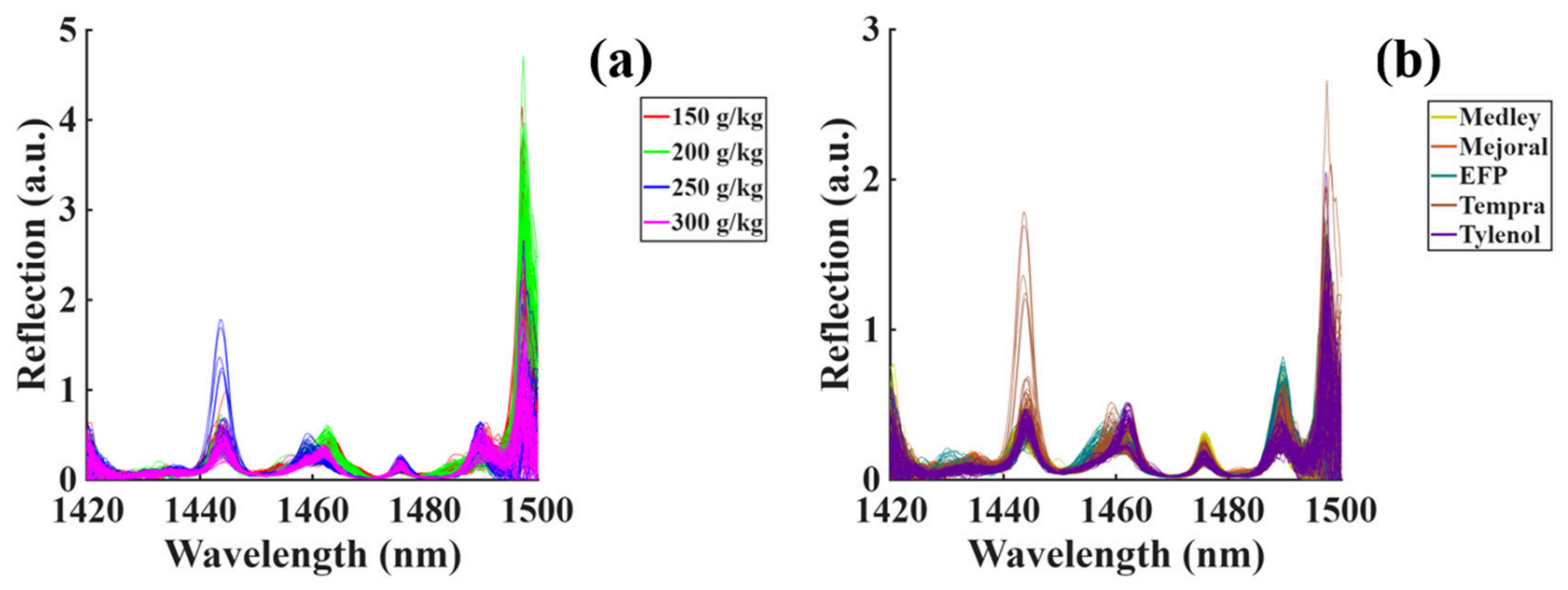
Refractometric sensor response. (**a**) Spectra response for Tempra paracetamol with different concentrations and (**b**) all brand spectra for 250 g/kg concentration.

Figure 11 shows an example of the output spectra for the Tempra brand sample at different paracetamol concentrations, together with the spectral responses of all brands at the same concentration. Figure 11a displays three measurements for each concentration over time, one per minute for 20 min (240 spectra). In the figure, some changes in the spectra can be seen as a function of concentration, which may be used to predict concentrations from a single spectrum; however, this task is not straightforward with these raw signals because it is not clear which information should be used since there are intensity variations and wavelength shifts. On the other hand, Figure 11b presents the sensor response spectra when exposed to a solution with a paracetamol concentration of 250 g/kg (240 spectra) and the corresponding excipient from all brands, as well as EFP (60 spectra) at the same concentration. From this figure, it can be observed that there are differences among the spectral profiles, suggesting the possibility of identifying the drug brand in the solvent. We can take advantage of this condition to recognize different paracetamol brands with the help of an ML-based classification algorithm.

**Figure 12:**
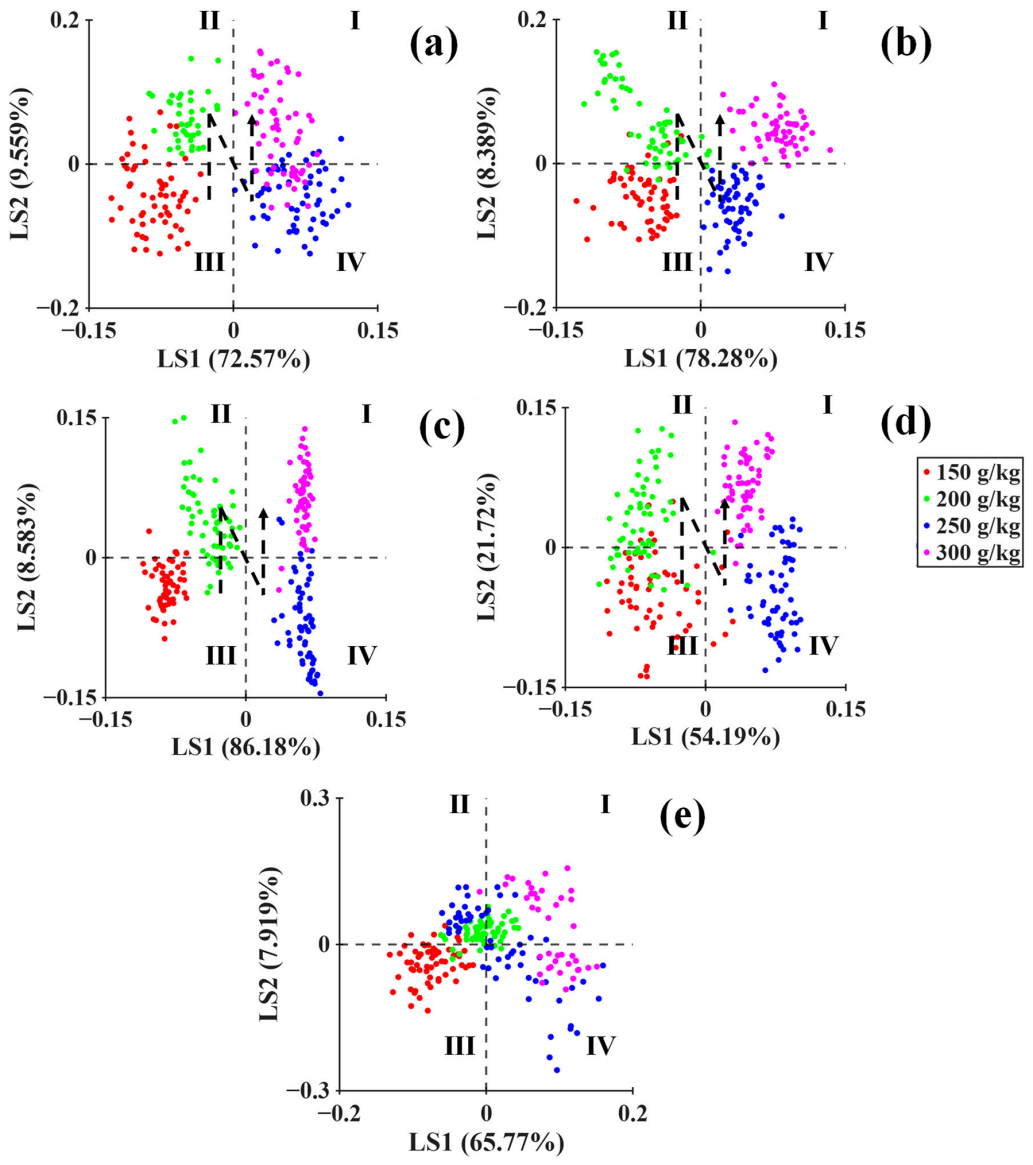
PLS using 2 LSs with different concentrations from 150 to 300 g/kg for the brands: (**a**) Medley, (**b**) Mejoral, (**c**) Tempra, (**d**) Tylenol and (**e**) EFP.

Before applying the classification algorithm, as previously mentioned, PLS was used as a dimensionality reduction technique to simplify the model and increase its robustness. In addition, a feature scaling (Min–Max) [34] process was performed to highlight the relevant information in the measured spectra. Figure 12 shows the PLS results using only two latent structures (LS1 and LS2) for each brand and for the different paracetamol concentrations, from the range of 150 to 300 g/kg; EFP is also shown. Each point on this figure corresponds to a complete spectrum, three different measurements of the same concentration, measured within 20 min, each minute (60 spectra for each concentration). As can be seen from the figure, in general, all commercial brands show a common trend: measurements with low concentrations (150 g/kg, red circles) predominantly start in the third quadrant (III), while at the next concentration level data (200 g/kg, green circles) are now in the second quadrant (II). This behavior is maintained at the following concentration values (250 and 300 g/kg, blue and magenta circles); they clustered together in the fourth (IV) and first quadrants (I), respectively. Figure 12e shows that PLS for the EFP does not follow this trend, which may be attributed to the crystallization of the dissolved drug on the surface of the sensor head, as will be discussed in the next paragraph.

**Figure 13:**
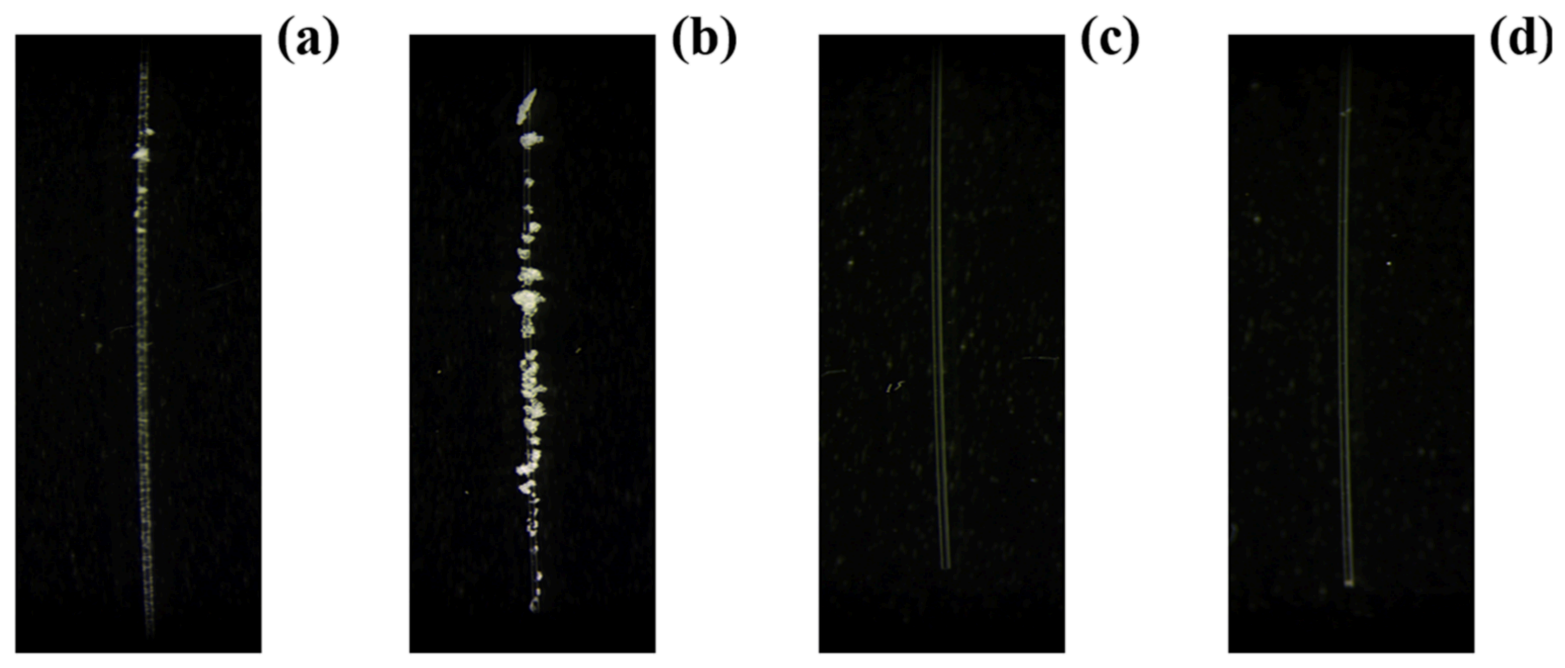
(**a**,**b**) Sensor head after 20 min in Tylenol and EFP 300 g/kg paracetamol solution, respectively. (**c**,**d**) Sensor head after rinsing with the control solution, respectively, showing crystallization removal.

Crystallization of paracetamol is quite a common phenomenon [8,9,10], Figure 13a,b show the sensor head after 20 min measurement in Tylenol and EFP solutions both at a concentration of 300 g/kg, respectively. Figure 13c,d show the sensor head section after 20 min rinsing with the control solution for both cases, where we can observe that the crystals were removed. From this figure, it can be noted that the main difference between measurements is the abundance of crystals, which is larger in the EFP case than in the Tylenol solution. We think that crystallization can decrease the contact area of the sensor head with the EFP solution, complicating the measurement and, as a result, its recognition.

**Figure 14:**
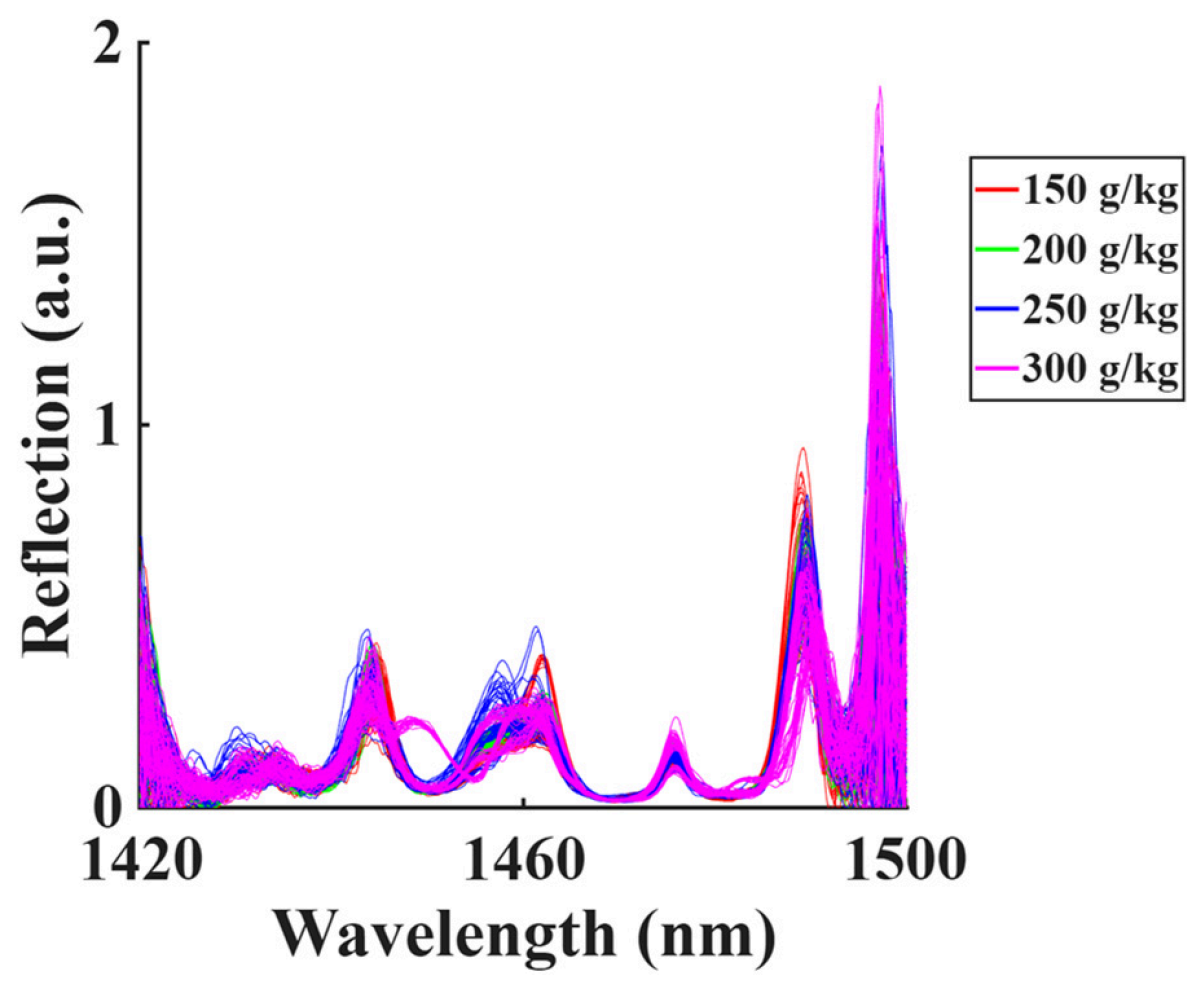
Raw data for the sensor response to the different EFP concentrations.

The complication in recognizing different concentrations because of the crystallization phenomenon can also be noticed from raw data of the sensor shown in Figure 14, where the spectra look similar for any concentration. However, as is shown in Figure 12e, PLS is still capable of distinguishing the different concentrations. However, we believe that this behavior is not relevant, since paracetamol is usually accessed through drugs that do not contain it in its pure form, and as will be seen later, even with this behavior, it is still possible to estimate its concentration in a suitable manner.

**Figure 15:**
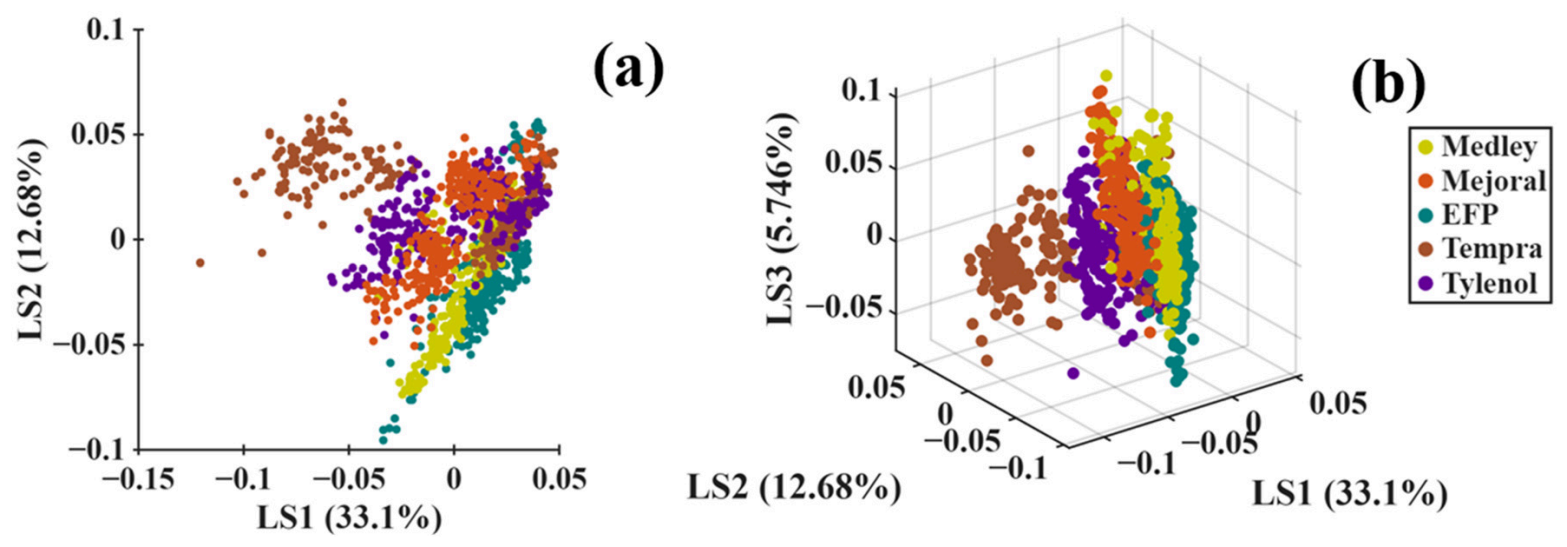
PLS for all brands and EFP using (**a**) 2 LSs and (**b**) 3 LSs.

Figure 15a shows the PLS for all paracetamol brands. This figure shows that there are clusters for each brand with partial overlaps, but they can still be recognized. Moreover, more LSs can be included to improve the classification. Figure 15b shows the PLS results with three LSs. This figure shows that brands with lower excipient content (see Table 2) are closer to EFP, while those with higher excipient content appear more separated. However, the Tempra brand data cluster is the farthest despite not having the highest amount of excipient. We think this is due to the nature of the excipient recipe used by this brand, which may not be like the other brands included in this study. Nevertheless, there was a notable improvement in the separation of the data clusters, although it is still possible to observe partial overlaps between each group, which makes it difficult to correctly classify each brand. It is still possible to further improve this separation by using a larger number of LSs; however, it is also possible to fall into overfitting, where the classifier correctly classifies the training data, but with new data (not present in the training set) performance could become poor. To determine the appropriate number of LSs, a cross-validation strategy was used in which two data sets were generated, one for training (measurement 1 and 2, 1600 spectra) and one for validation (measurement 3, 800 spectra), using k-fold strategy (*k* = 5) and a gaussian kernel with auto-scale in the SVM training, which means training the model with 80% of the data and performing validation with the remaining 20%. This process was performed with the latent structures obtained with the PLS over all the experimental spectra, starting with one LS until 10 LSs, increasing one by one and recording the F1-score each time.

**Figure 16:**
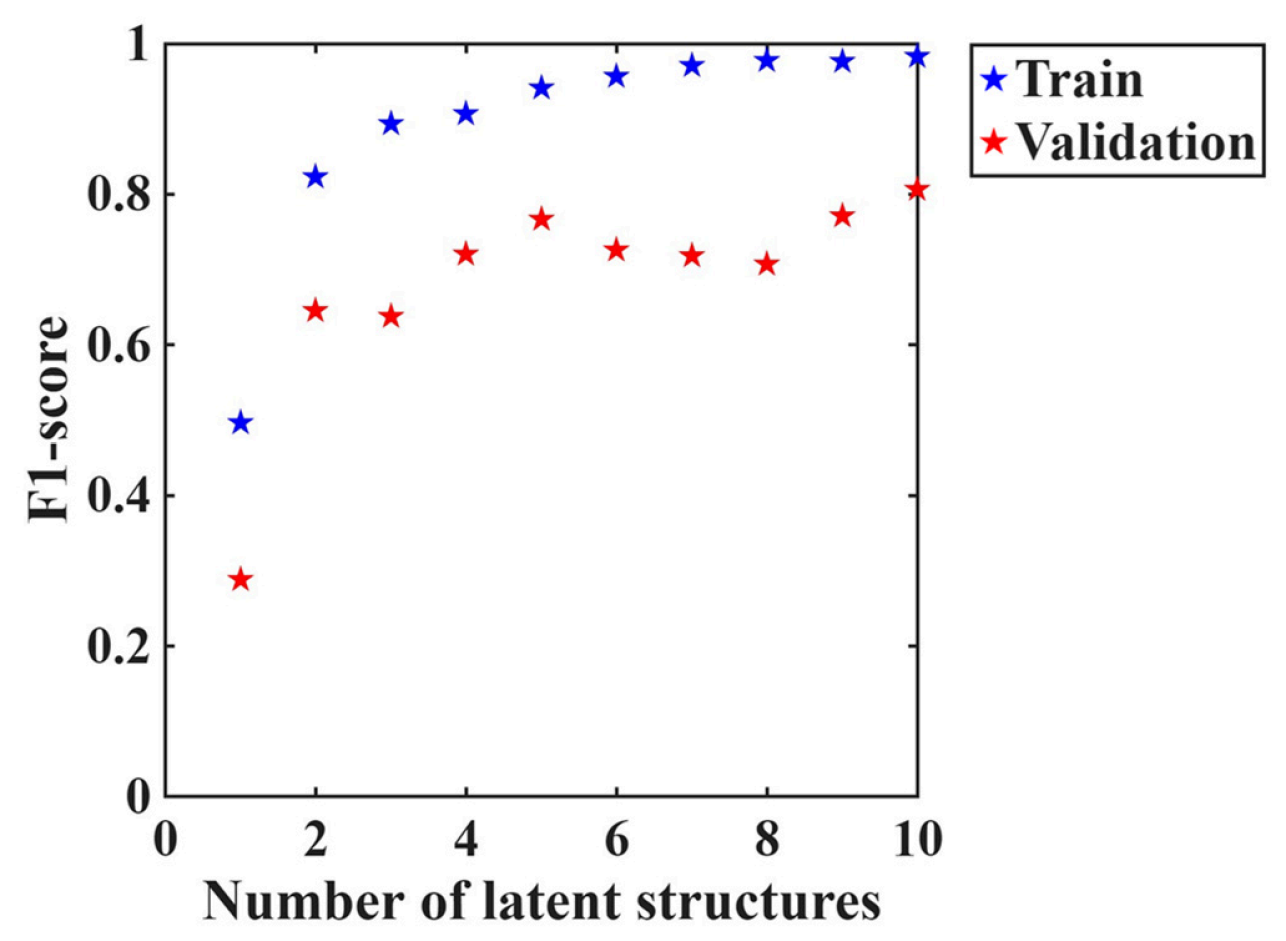
Learning curve of the SVM classifier (F1-score) as a function of the number of LSs.

Figure 16 shows the evolution of the SVM model performance (learning curve) for the F1-score of the training and validation data as the number of LSs increases. The *x*-axis shows the number of LSs used, while the *y*-axis shows the average F1-score calculated from the confusion matrix at each stage of the learning curve. This graph shows that, starting with the fourth LS, the validation F1-score no longer changes significantly, even when this value continues increasing in the training set. Thus, the optimal number of LSs to use was four, since using a larger number of LSs could potentially lead to overfitting.

**Figure 17:**
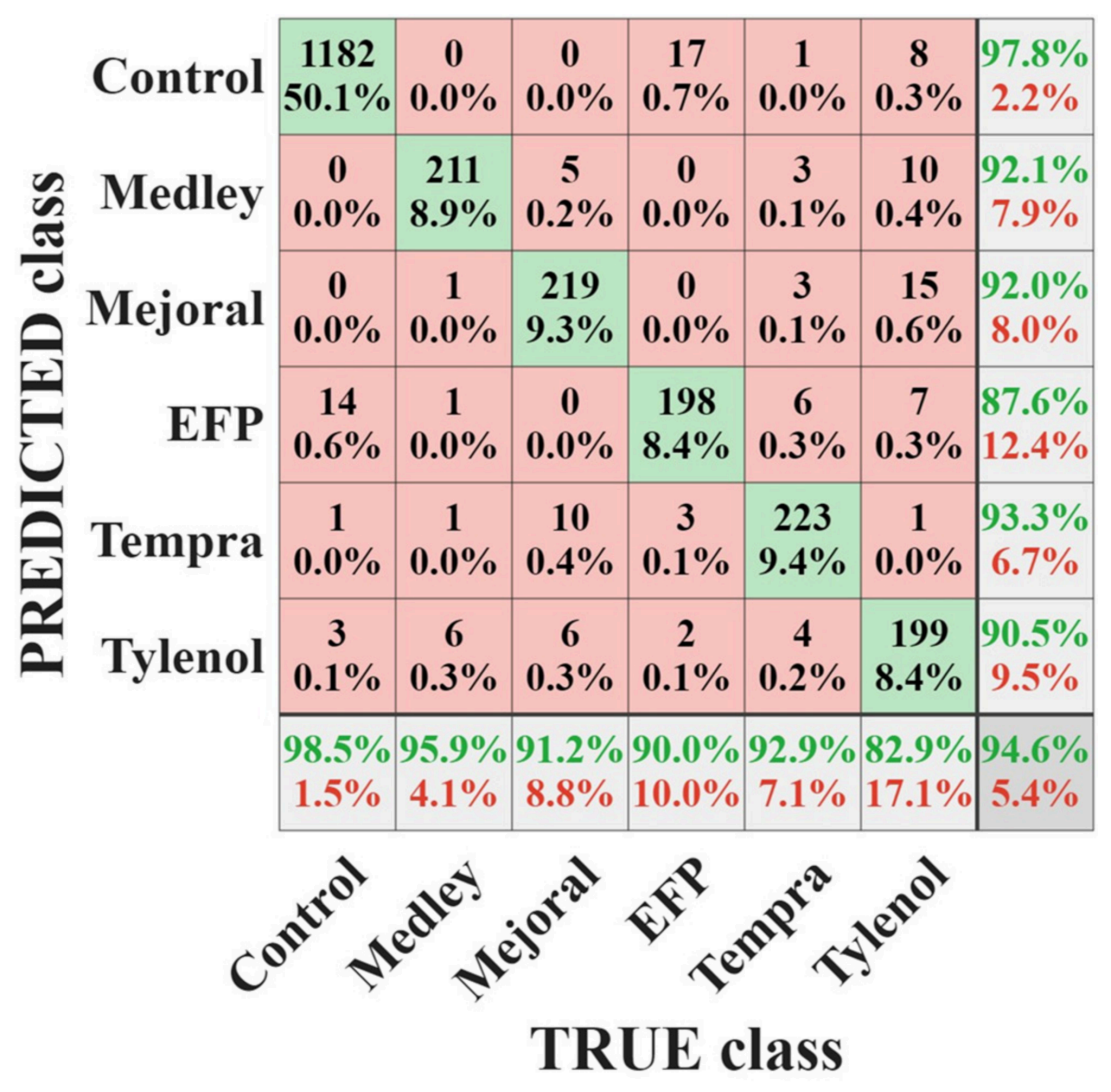
Multivariable confusion matrix using four LSs for the trained SVM to recognize the four paracetamol brands, EFP and control.

Figure 17 shows the results of the confusion matrix for the SVM model using four LSs. The model was trained to identify six different classes: Control, Medley, Mejoral, EFP, Tempra, and Tylenol. The SVM was trained with a Gaussian kernel with auto-scaling and k-fold cross-validation with *k* = 5. The figure shows two numbers in each square; the numbers above represent the total number of correct or incorrect classifications of the measurements, while the numbers below are the percentages relative to the total number of trained measurements. On the other hand, the percentages in the last column correspond to the precision for each class (percentage in green) and the false discovery rate (percentage in red). The information in the last row corresponds to the recall metric (green) and the false negative rate (red).

The results showed that for the control, 1182 predictions were made correctly (representing a precision of 97.8%, while the predictions for Medley, Mejoral, Tempra, Tylenol, and EFP were 211, 219, 223, 199, and 198 correct predictions (92.1%, 92%, 93.3%, 90.5%, and 87.6%) precision, respectively. Table 3 shows a summary of the classifier performance metrics. The Medley brand was the best (highest F1-score) in the classification (94%) among those measurements with the paracetamol/excipient mixture, while the Tylenol brand obtained the lowest F1-score (86.5%), which contains the highest concentration of excipient. On the other hand, EFP had an F1-score of 88.8%, which is quite low, probably due to the crystallization effect of paracetamol, as was mentioned before. In general, the performance of the multi-class SVM classifier has a global F1-score of 92% when identifying between different brands of paracetamol, EFP and control signals.

**Figure 18:**
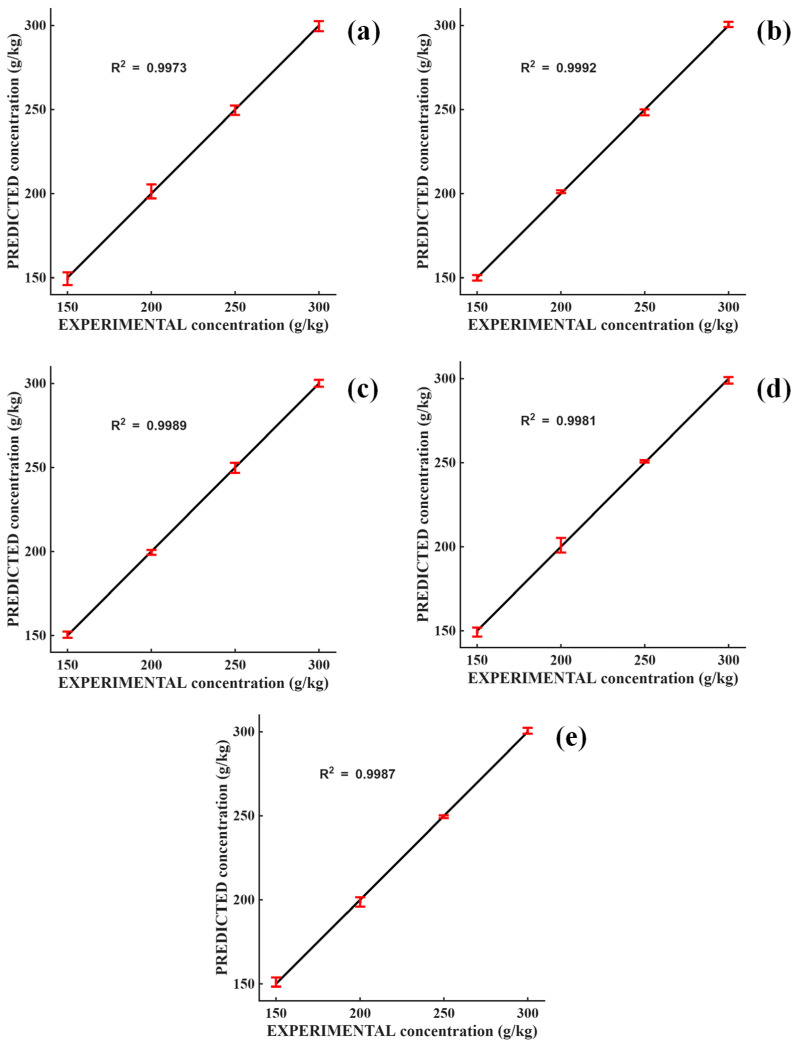
PLSR of concentration using three LSs for (**a**) Medley, (**b**) Mejoral, (**c**) Tempra, (**d**) Tylenol and (**e**) EFP.

Once the classification task (brand identification) has been completed, it was possible to quantify the concentration of paracetamol and/or excipient present in the solution using PLSR. Figure 18 shows the relationship between experimental concentrations and the predicted concentrations using the PLSR model with only three LSs (one less LS was chosen to further reduce possible overfitting). The proximity of the points to the ideal prediction line showed high predictive power, accompanied by low dispersion with $R^{2}$ values above 0.99. These regressions were performed using data from minute 20th, when it was considered that the system had reached steady state. One parameter to evaluate the performance of the sensors is LOD, which was defined using the standard deviation between the experimental concentration values and the predicted ones (assuming SNR = 1). From these graphs, the regression for the Mejoral brand data had the lowest LOD (1.58 g/kg), while the Medley brand had the highest LOD (2.93 g/kg). In any case, the results showed a high resolution of the sensor for the different brands analyzed.

**Figure 19:**
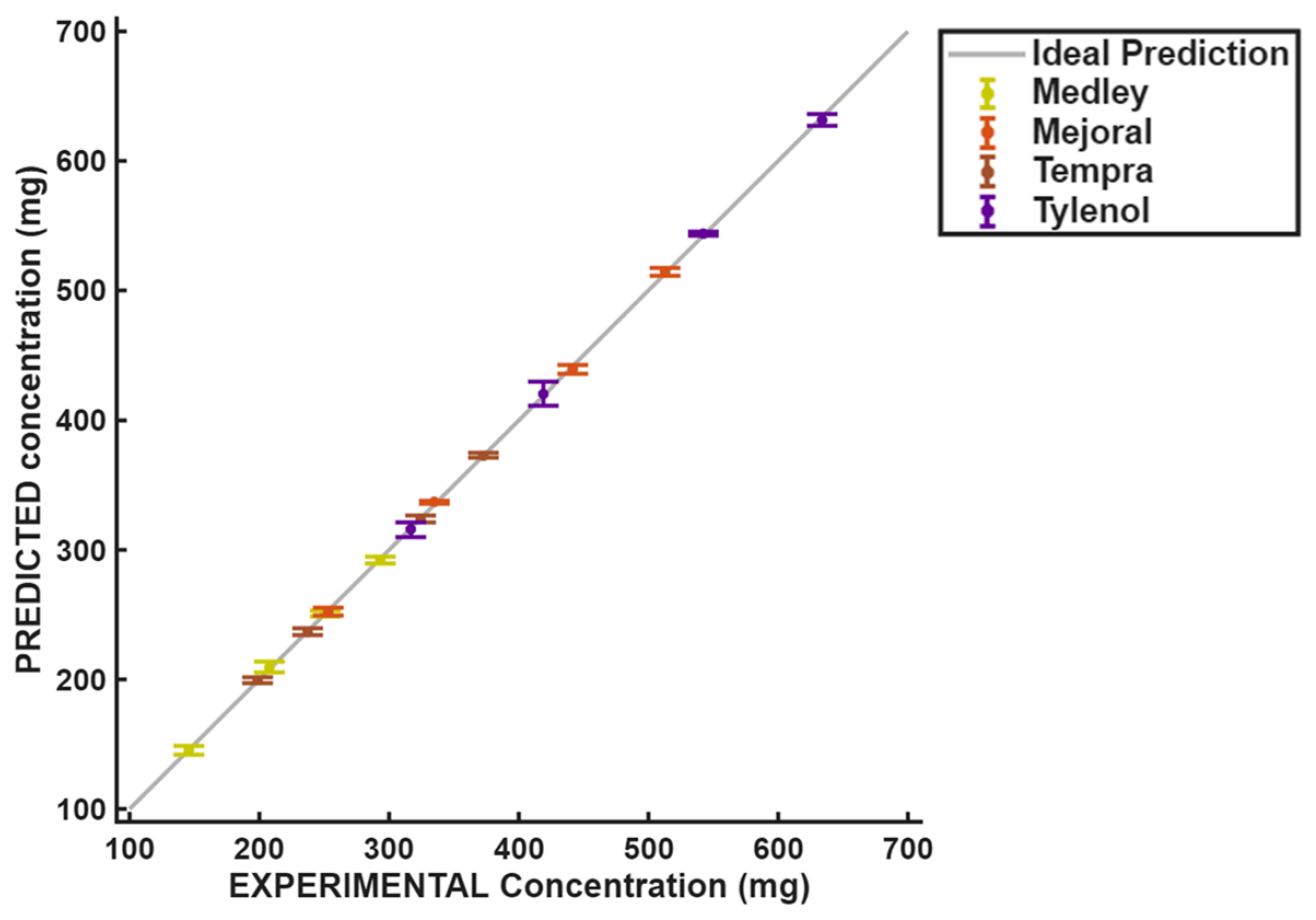
PLSR of the excipient using three LSs for the four paracetamol brands. The solid line represents the ideal prediction.

On the other hand, Figure 19 shows the comparison between the experimental excipient content and that predicted by the PLSR model, again using three LSs. As in the concentration analysis, the model was evaluated considering only the data at minute 20. For simplicity, all measurements are shown in a single graph. The regression model with the best LOD in the estimation of the excipient by brand was for Tempra (1.93 mg), while the highest was for Tylenol (5.12 mg). In any case, the results showed that the proposed sensor could not only estimate the concentration of paracetamol but was also able to predict the amount of excipient present in each solution.

Table 4 shows a summary of the regression models for predicting paracetamol and excipient concentrations by brand, as well as the corresponding LOD. In all cases, the first three LSs were used.

## 4. Conclusions

In this work, a refractometric sensor was developed based on four cascaded Michelson interferometers with LPFGs of different periods (450, 475, 500, and 525 μm). The spectral responses of the sensor were analyzed with PLS for dimension reduction. SVM algorithm and PLSR were used to determine the brand and paracetamol/excipient content of four commercial brands of paracetamol: Medley, Mejoral, Tempra, and Tylenol, respectively, and the paracetamol concentration of EFP solutions. The results obtained showed that it was possible to determine the brand of the medication with an overall F1-score performance of the SVM classifier of 92%. On the other hand, the estimation of the concentration of paracetamol with the PLSR models for the brands had LODs of 2.93, 1.58, 1.85, and 2.45 g/kg, respectively, while for the excipient concentrations the LODs were 2.75, 2.99, 1.93, and 5.12 mg, respectively. The best LODs were Mejoral and Tempra for paracetamol and excipient concentration, respectively.

In addition, the results showed that the proposed method was sensitive to differences in composition between brands. We can also mention that the spectral response generated by the sensor contained sufficient information for both recognition and quantitative prediction. The use of multiple LPFG-based cascade interferometers is an innovative aspect of the work, as it increased the spectral complexity of the response and thus enriched the features available for ML-based analysis. Moreover, the study supports the potential of combining refractometric sensing with fiber optic and ML techniques for pharmaceutical analysis.

Finally, the standard pharmaceutical quality control methods, such as weight variation, content uniformity, and solution testing, could be complemented by the proposed sensor as a useful tool for preliminary analysis and discrimination between brands. On the other hand, compared to chromatographic techniques such as HPLC or conventional spectroscopic methods such as UV-Visible spectroscopy, the refractometric sensor has potential advantages in terms of operational simplicity and relatively shorter measurement times.

## Figures and Tables

**Table 1:** Metrics for class $C_{i}$ and global metrics of the SVM model.

Metrics per Class	Equation	Global Metrics	Equation
${Recall}_{C_{i}}$	$\frac{TP_{C_{i}}}{TP_{C_{i}}+FN_{C_{i}}}$	Recall (macro)	$\frac{1}{K}\sum_{i=1}^{K}{Recall}_{C_{i}}$
${Precision}_{C_{i}}$	$\frac{TP_{C_{i}}}{TP_{C_{i}}+FP_{C_{i}}}$	Precision (macro)	$\frac{1}{K}\sum_{i=1}^{K}{Precision}_{C_{i}}$
${Accuracy}_{C_{i}}$	$\frac{TP_{C_{i}}+TN_{C_{i}}}{TP_{C_{i}}+TN_{C_{i}}+FP_{C_{i}}+FN_{C_{i}}}$	Accuracy	$\frac{\sum_{i=1}^{K}TP_{C_{i}}}{\sum_{i=1}^{K}\sum_{j=1}^{K}C_{i,j}}$
${Specificity}_{C_{i}}$	$\frac{TN_{C_{i}}}{TN_{C_{i}}+FP_{C_{i}}}$	Specificity (macro)	$\frac{1}{K}\sum_{i=1}^{K}{Specificity}_{C_{i}}$
${F1-score}_{C_{i}}$	$2\cdot \frac{\;{Precision}_{C_{i}}\;\cdot \;{Recall}_{C_{i}}}{{Precision}_{C_{i}}+{Recall}_{C_{i}}}$	F1−score (macro)	$\frac{1}{K}\sum_{i=1}^{K}{F1-score}_{C_{i}}$

**Table 2:** Mass used in each solution for each commercial pharmaceutical brand of paracetamol.

Brand	Concentration (g/kg)	$m_{i}$ (mg)	API (mg)	Excipient (mg)
Medley	150	1646	1500	146
	200	2208	2000	208
	250	2751	2500	251
	300	3293	3000	293
Mejoral	150	1753	1500	253
	200	2335	2000	335
	250	2942	2500	442
	300	3513	3000	513
Tempra	150	1699	1500	199
	200	2237	2000	237
	250	2824	2500	324
	300	3373	3000	373
Tylenol	150	1817	1500	317
	200	2419	2000	419
	250	3042	2500	542
	300	3634	3000	634

**Table 3:** Class and global metrics of the classifier using four LSs.

Class	Precision	Recall	Specificity	Accuracy	F1-Score
Control	97.8%	98.5%	97.8%	98.1%	98.2%
Medley	92.1%	95.9%	99.2%	98.9%	94.0%
Mejoral	92.0%	91.3%	99.1%	98.3%	91.6%
EFP	87.6%	90.0%	98.7%	97.9%	88.8%
Tempra	93.3%	92.9%	99.2%	98.6%	93.1%
Tylenol	90.5%	82.9%	99.0%	97.4%	86.5%
Global	92.2%	91.9%	98.8%	94.6%	92.0%

**Table 4:** Regression models for paracetamol and excipient concentration. The LOD for each model by brand is shown.

Brand	Concentration Model Regression ($y_{c}=w_{0}^{c}+w_{1}^{c}\cdot LS1+w_{2}^{c}\cdot LS2+w_{3}^{c}\cdot LS3$)	LOD (g/kg)
Medley	$y_{c}=225+181.81\cdot LS1+61.50\cdot LS2+23.64\cdot LS3$	2.93
Mejoral	$y_{c}=225+185.58\cdot LS1+53.56\cdot LS2+12.73\cdot LS3$	1.58
EFP	$y_{c}=225+178.60\cdot LS1+71.68\cdot LS2+20.40\cdot LS3$	1.99
Tempra	$y_{c}=225+187.07\cdot LS1+47.41\cdot LS2+14.67\cdot LS3$	1.85
Tylenol	$y_{c}=225+178.47\cdot LS1+71.53\cdot LS2+21.42\cdot LS3$	2.45
	**Excipient Model Regression** ($y_{e}\;=\;w_{0}^{e}\;\;+w_{1}^{e}\cdot LS1+w_{2}^{e}\cdot LS2+w_{3}^{e}\cdot LS3$)	**(mg)**
Medley	$y_{e}=224.5+177.34\cdot LS1+58.74\cdot LS2+22.31\cdot LS3$	2.75
Mejoral	$y_{e}=385.75+330.52\cdot LS1+93.52\cdot LS2+23.89\cdot LS3$	2.99
EFP	NA	NA
Tempra	$y_{e}=283.25+231.36\cdot LS1+54.83\cdot LS2+16.76\cdot LS3$	1.93
Tylenol	$y_{e}=478+383.88\cdot LS1+154.31\cdot LS2+44.83\cdot LS3$	5.12

## Data Availability

The data sets generated during and/or analyzed during the current study are available from the corresponding author on reasonable request.

## Abbreviations

The following abbreviations are used in this manuscript:

EFP	Excipient-free paracetamol
SVM	Support vector machine
PLSR	Projection to latent structures regression
LOD	Limit of detection
API	Active pharmaceutical ingredient
LPFG	Long-period fiber grating
ML	Machine learning
PLS	Projection to latent structures
LS	Latent structures
TP	True positive
TN	True negative
FP	False positive
FN	False negative
OSA	Optical spectrum analyzer
SLD	Superluminescent diode
